# Toward Sustainable Wax Extraction from the *Saccharum officinarum* L. Filter Cake Byproduct: Process Optimization, Physicochemical Characterization, and Antioxidant Performance

**DOI:** 10.1021/acssuschemeng.3c03279

**Published:** 2023-08-30

**Authors:** Francisca S. Teixeira, Paula T. Costa, Susana S. Vidigal, Manuela Pintado, Lígia L. Pimentel, Luís M. Rodríguez-Alcalá

**Affiliations:** CBQF—Centro de Biotecnologia e Química Fina—Laboratório Associado, Escola Superior de Biotecnologia, Universidade Católica Portuguesa, Rua Diogo Botelho 1327, 4169-005 Porto, Portugal

**Keywords:** sugarcane, filter cake, antioxidant, extraction, lipids, ethanol, residues, circular economy, GC−MS

## Abstract

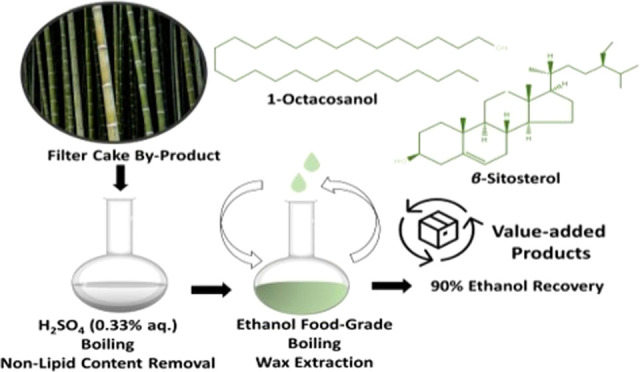

*Saccharum officinarum* L. exploitation and processing result in different byproducts, such as filter cake (FC). This study aimed to establish the most suitable experimental conditions to obtain lipophilic bioactive compounds from FC industrial residues, considering their high efficiency, cost-effectiveness, extraction yield, composition, and physicochemical properties. Results indicated that the most appropriate methodology consisted of the pretreatment of the FC sample with H_2_SO_4_, followed by ethanolic extraction (B6 method), avoiding energy-consumption FC drying steps and providing ethanol recovery (approx. 90%). The obtained B6 extract yield was 9.59 ± 0.27 g/100 g of FC dry weight, and this methodology proved to be more efficient in obtaining fatty alcohols (20.28 ± 1.48 g/kg extract) and phytosterols (31.56 ± 0.18 g/kg extract) while maintaining lower total monosaccharide concentration (26.19 ± 1.82 mg/g extract). Furthermore, the geographically related multivariate analysis in wax composition and antioxidant activity was evaluated by comparing B6 waxes from Guariba (G) and Univalem (U), both provided by Brazil and collected in June 2020. Overall, the wax composition is affected, but the antioxidant activity is uncompromised, which indicates that the optimized wax extraction method can be applied to FC.

## Abstract 2

Ethanolic extraction of the filter cake industrial byproduct supports the implementation of circular economy methodologies applied to the research and development of sustainable lipophilic materials.

## Introduction

Filter cake (FC) is a byproduct that results from the processing of *Saccharum officinarum* L. (sugarcane). It is a slurry that emerges from sugar and ethanol production industries and is currently used as fertilizer in sugarcane fields due to its abundance in minerals and organic matter.^1,2^ Furthermore, as sugar is produced by extracting the juice from crushed sugarcane, several byproducts besides filter cake including sugarcane straw, bagasse, and vinasse are released during this process.^1−3^

With a global sugarcane production of 72.91 tons/ha, the top producers contribute to approximately 1.75 billion tons annually, Brazil being the main leader in the manufacture of ethanol sector.^4^ Brazil and other countries such as Pakistan are increasing sugarcane production, making the filter cake industrial output 2.7 million tons during 2022/2023.^3,5^ An important environmental and economic concern is how to dispose of filter cake, since it represents an approximate production of 30 kg/ton of milled sugarcane.^4,6^ Currently, most sugarcane waste (i.e., filter cake, straw, and bagasse) biomass is burned, unused, or discarded, and it is crucial to adopt innovative circular economy policies in the sugarcane industrial sector as a key strategy to reduce, reuse, and recycle these generated biowastes.^5,7^

Unprocessed filter cake contains approximately 75% of water, and its composition depends on several factors: type of soil, sugarcane variety, harvesting method, juice extraction, and other products used for clarification and filtration methods. Furthermore, the filter cake can be used to produce bio-wax as an alternative to other vegetable, animal, and synthetic waxes. Such materials can be applied to pharmaceutical (i.e., anti-obesity, hypocholesterolemic, and antioxidant agents), chemical (i.e., coating applications, cleaning, and polishing), or cosmetic (i.e., thickening lipstick) industrial fields.^6,8^

Previous studies identified long-chain aliphatic primary alcohols (policosanol), phytosterols (stigmasterol, β-sitosterol), triterpenoids, wax esters, aldehydes, and fatty acids in sugarcane-derived waxes.^1,9^ Thus, phytosterols are valued as antioxidants due to their free radical scavenger capacity.^10^ Moreover, the hypocholesterolemic effects of phytosterols and the anti-obesity properties of octacosanol have also been evidenced in previous studies.^11,12^ In addition, other less-apolar compounds such as phenolic acids, anthocyanins, and carotenoids can be present in vegetable plants. Usually, they are involved in plant adaptation to adverse environmental conditions and are considered antioxidant agents.^13^

Previous efforts in the chemical and engineering sector are being made in order to prevent the utilization of petroleum solvents such as hexane, dichloromethane (DCM), benzene, and toluene, which are highly volatile extractants that contribute to climate change, pollution, and hazard manufacturing health problems.^14^ Greener/sustainable procedures that bypass the handling of these solvents should be preferentially chosen.^15^ Bio-based ethanol from sugarcane biomass feedstock is classified as a biodegradable and low-toxicity solvent, and it has proven its cost-effectiveness and high efficiency in extracting target bioactive compounds.^16^ Boiling condensation extraction cycles can improve extraction yields and lead to fewer solvent requirements.^15^ The assessment of extraction parameters such as time and temperature is crucial, as they greatly influence the effective yields of the bioactive molecules in extracts.^17^

During the first decade of the XXI century, the high requirement for sugar and ethanol production led to the expansion of sugarcane crops in Brazil. The concentration of sucrose, resistance to pests, and water stress are some varying factors of the local climate, soil type, and chemical, physical, and morphological attributes.^19^ Generally, epicuticular wax compounds [that can be free fatty acids (FFAs), primary alcohols, alkanes, and aldehydes] increase in plants in response to abiotic stress (e.g., deficient or excessive water).^20^

This study aimed to evaluate the optimal filter cake wax isolation conditions, using boiling methods to contribute to the valorization of this waste stream. Seven different ethanolic methods were used to extract wax from the filter cake byproduct attending to different pretreatment, extraction temperature, and solvent mixtures. After the selection of the better-suited extraction method, the geographically related variation in extracts was assessed within two different batches of raw filter cake provided from different *S. officinarum* L. crops in Brazil that are approximately 300 km apart, namely, Guariba and Univalem, both collected in June 2020.

## Materials and Methods

### Materials and Chemicals

Sugarcane filter cake was provided by Raízen (Brotas, Brazil) from different *S. officinarum* L. crops in Brazil (Guariba and Univalem). For the lipid extraction optimization, the Guariba batch collected in November 2019 was used, and for the study of geographical variation, Guariba and Univalem batches both collected in June 2020 were used.

Ethanol (EtOH, F.C.C. Food Grade 96% v/v) was purchased from PanReac AppliChem (Barcelona, Spain). For boiling pretreatment, tap water was used and, when required, acidified with sulfuric acid (H_2_SO_4_, 95.0–98.0%) purchased from Merck (Darmstadt, Germany).

For high-performance liquid chromatography (HPLC) analysis, 2-propanol (LC–MS Grade ≥99.9%), isooctane (HPLC Grade ≥99.8%), and dichloromethane (DCM) (HPLC Grade ≥99.9%) were purchased from VWR Chemicals (Radnor, Pennsylvania); acetone (HPLC Grade ≥99.8%) from Thermo Fisher Scientific (Waltham, Massachusetts), ethyl acetate (HPLC Grade ≥99.7%) and water (for HPLC, analytical grade) from Honeywell (Charlotte, North Carolina), acetic acid (HPLC Grade ≥99.8%) from Carlo Erba Reagents (Barcelona, Spain), and triethylamine (TEA) (≥99.5%) from Merck (Darmstadt, Germany).

For gas chromatography–mass spectrometry (GC–MS) analysis, the analytical standard tetracosane (99%) and the derivatizing reagent *N*,*O*-bis(trimethylsilyl)trifluoroacetamide with 1% trimethylchlorosilane (BSTFA) were purchased from Merck (Darmstadt, Germany).

For neutral monosaccharide quantification, samples were treated with sodium borohydride (NaBH_4_), ammonia solution (NH_3_, 32%), 1-methylimidazole (99%), acetic anhydride (99%), anhydrous acetone (99.9%), and 2-deoxy-d-glucose (≥98%), obtained from Merck (Darmstadt, Germany).

During the antioxidant experiments, phosphate buffer solution (75 mM, pH 7.4) was prepared by using the salt solution of sodium dihydrogen phosphate (NaH_2_PO_4_) anhydrous, ≥98%, purchased from Merck (Darmstadt, Germany) and Trolox (6-hydroxy-2,5,7,8-tetramethylcroman-2-carboxylic acid; 97%) obtained from Merck (Darmstadt, Germany).

DPPH^•^ (2,2′-diphenyl-1-picrylhydrazyl, 95%), from Thermo Fisher Scientific (Waltham, Massachusetts), ABTS [2,2′-azino-bis(3-ethylbenzothiazoline-6-sulfonic acid)] (≥98%), potassium persulfate (K_2_O_8_S_2_, ≥99.0), and 2,2′-azo-bis-(2-methylpropionamidine)-dihydrochloride (AAPH, 97%) were purchased from Merck (Darmstadt, Germany) as well as the fluorescein disodium salt. For total phenolic content determination, Folin–Ciocalteu’s phenol reagent (FCr) and sodium carbonate (Na_2_CO_3_, 99%) were obtained from Merck (Darmstadt, Germany).

The human keratinocyte cell line HaCaT was purchased from CLS—Cell Line Services (no 300493), and Dulbecco’s modified Eagle medium from Thermo Fischer (Waltham, Massachusetts, EUA) supplemented with 10% fetal bovine serum (FBS) (Thermo Fischer, Waltham, Massachusetts, EUA) and 1% penicillin–streptomycin antibiotic (Thermo Fischer, Waltham, Massachusetts, EUA). For cytotoxicity, the PrestoBlue assay (Thermo Fischer, Waltham, Massachusetts, EUA) was used. For cell culture, dimethyl sulfoxide (DMSO, molecular biology grade) was used and purchased from Merck (Darmstadt, Germany).

### Lipid Extraction

**Figure 1:**
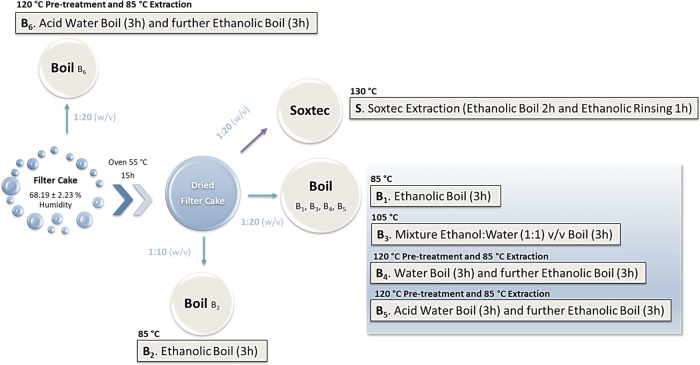
Schematic representation of the used lipid extraction methods (S; B1–B6).

Filter cake samples were subjected to different ethanolic lipid extraction conditions, Soxtec (S) and Boiling (B), varying sample pretreatment conditions: i.e., water boiling or H_2_SO_4_ (aq.) 0.33% w/v solution boiling; extraction temperature (85, 105, or 130 °C), and sample/solvent proportion (1:10 or 1:20 w/v). Further, depending on the extraction method, filter cake samples were dried at 55 °C on a Thermo Fisher Scientific Oven (Waltham, Massachusetts) until constant weight (15 h). A summary of the assayed isolation methods is depicted in Figure 1 and Table 1.

**Table 1:** Conditions of Lipid Extraction Methods^a^

method	pretreatment	extraction solvent	sample/solvent ratio (w/v)	temperature (°C)	extraction time (h)
**S**	no	EtOH	1:20	130	2 h boil + 1 h rinsing
**B**_**1**_	no	EtOH	1:20	85	3 h boil
**B**_**2**_	no	EtOH	1:10	85	3 h boil
**B**_**3**_	no	EtOH/H_2_O 1:1 (v/v)	1:20	105	3 h boil
**B**_**4**_	boiling (H_2_O)	EtOH	1:20	120/85	3 h boil/3 h boil
**B**_**5**_	boiling (H_2_SO_4_ (aq.) 0.33% w/v)	EtOH	1:20	120/85	3 h boil/3 h boil
**B**_**6**_	boiling (H_2_SO_4_ (aq.) 0.33% w/v)	EtOH^b^	1:20	120/85	3 h boil/3 h boil

a S: Soxtec; B: Boiling.

b Before and after pretreatment, the filter cake was not dried.

In the procedure labeled as S method and used as a reference, the lipophilic extraction was carried out in triplicate using a Foss SoxtecTM 8000 apparatus (Hilleroed, Denmark). Within this method, previously dried FC samples were extracted with EtOH at the proportion of 1:20 (w/v) at 130 °C for 2 h of boiling followed by 1 h of rinsing at atmospheric pressure. Afterward, the resultant extraction solution was evaporated in a rotary evaporator Heidolph HeiVAP (Schwabach, Germany) under reduced pressure (gradually decreased to 50 mbar) in a temperature-controlled bath at 40 °C.

The boiling methods identified as B1–B5 were performed using previously dried FC, while the B6 method was performed using wet FC (about 68.19% humidity). Moreover, boiling methods B1–B3 were performed without FC pretreatment, while B4 included a pretreatment procedure with water, and B5 and B6 involved a pretreatment with a H_2_SO_4_ (aq.) 0.33% w/v solution.

In general, these methods consisted of the mixing of FC and solvent at different proportions in a 500 mL round bottom flask that was kept under reflux in a silicone oil bath at different temperatures with constant magnetic stirring for different time periods [3 h (without pretreatment) or 6 h (including FC pretreatment)]. Afterward, the mixture was filtered while still hot through a synthetic fabric filter (Tescoma) using a porcelain Büchner funnel under vacuum and then evaporated (as described for the Soxtec method).

Extraction methods B1 and B2 were performed using the same extraction solvent, EtOH, at the same temperature (85 °C), only varying the sample/solvent (w/v) proportion, which was 1:20 (w/v) for B1 and 1:10 (w/v) for B2. Within the B3 method, a mixture of ethanol/water at 1:1 (v/v) was used as extraction solvent; the sample/solvent ratio was 1:20 (w/v) and the temperature was set at 105 °C. Regarding boiling methods B4–B6, all included an FC pretreatment that consisted of previous aqueous extraction of the FC samples before their ethanolic extraction. In a 500 mL round bottom flask, the FC samples were mixed with water (B4) or a H_2_SO_4_ (aq.) 0.33% w/v solution (B5 and B6) at a sample/solvent proportion of 1:20 (w/v), placed in a silicone oil bath at 120 °C, under reflux and constant magnetic stirring for 3 h. After that, the mixture was filtered as described above, and the obtained filter cake dried at 55 °C for 15 h (except for method B6 in which FC was not dried after the pretreatment). Finally, the pretreated FC was extracted with ethanol under the same conditions as B1.

In the end, all of the obtained extracts (S and B1–B6 methods) were completely dried, until constant weight, in a vacuum oven at 60 °C and 100 mbar and then weighed, and the respective yields were assessed.

### Neutral Monosaccharide Determination-Gas Chromatography-Flame Ionization Detection (FID) Analysis

The quantification of neutral monosaccharides (2-deoxy-2-ribose, l-rhamnose, fucose, d-ribose, arabinose, xylose, mannose, galactose, and glucose) was performed by polysaccharide reduction and acetylation in alditol acetates according to Pinto et al.^21^ and Faustino et al.^22^ Therefore, 2–3 mg of the sample was accurately weighed into glass tubes, and polysaccharides were hydrolyzed with 2 M H_2_SO_4_ before the derivatization reaction. Monosaccharides were reduced with NaBH_4_ (15% in NH_3_ 3 M) and then acetylated with acetic anhydride in the presence of 1-methylimidazole. The mixture was centrifuged for phase separation, the aqueous phase was removed, and analytes were separated and detected by GC-FID (Agilent Technologies, 7890B model) using a DB-225 (J&W Scientific, Folsom, CA) capillary column (30 m length, 0.25 mm diameter, 0.15 μm thickness). For the quantification of neutral monosaccharides, 2-deoxyglucose (200 μL at 2 mg/mL) was used as an internal standard.

### Gas Chromatography Triple Quadrupole Mass Spectrometry (GC–MS)

For the analysis by GC–MS, samples were derivatized into their trimethylsilyl (TMS) derivatives. In a glass vial, samples were accurately weighed (5 mg), and 100 μL of tetracosane/internal standard (0.5 mg/mL in DCM), 30 μL of BSTFA, and DCM to a final volume of 1.3 mL was added. The mixture was incubated for 60 min at 30 °C. The derivatized samples were analyzed on a GC–MS model EVOQ (Bruker, Karlsruhe, Germany) coupled to a mass spectrometer, with a Rxi-5Sil MS column (30 m × 250 μm × 0.25 μm) at a constant flow of 1 mL/min. The carrier gas used was helium, and the GC–MS conditions were as described by Teixeira et al.^18^ The injector temperature was set at 330 °C with a split of 10, and the oven temperature started at 60 °C with a hold for 5 min, increasing at a rate of 3 °C/min until 330 °C and maintained for 20 min. The MS detector was operated in electron ionization (EI) mode at 70 eV, a source temperature of 280 °C, a transfer line at 300 °C, and a quadrupole in a scan range from 33 to 1000 amu per second. The identification was based on the comparison of the obtained mass spectra with the information on the NIST Library (v.2.3).

### High-Performance Liquid Chromatography-Evaporative Light Scattering Detection (HPLC-ELSD)

The samples were prepared to a concentration of 3 mg/mL in DCM and afterward analyzed on an HPLC (Model 1260 Infinity II; Agilent Technologies, Santa Clara, CA) attached to an Evaporative Light Scattering Detector (ELSD; 1290 Infinity II, Agilent Technologies, Santa Clara, CA) using nitrogen as the nebulizing gas coupled to a Zorbax RX-SIL column (2.1 × 150 mm, 5 μm; Agilent Technologies, Santa Clara, CA). Analysis conditions were assayed as described by Abreu et al.,^23^ with some modifications described by Teixeira et al.^18^ The flow rate was set at 0.275 mL/min with an injection volume of 20 μL and the detector evaporator and nebulizer temperature was set at 60 °C with nitrogen as the nebulizing gas at a 1.20 SLM flow rate. In all performed analyses, all of the samples were injected at least in triplicate.

### Fourier-Transform Infrared Spectroscopy with Attenuated Total Reflectance (FTIR-ATR)

The samples were analyzed on a PerkinElmer Paragon 1000 FTIR (Waltham, Massachusetts) with the ATR accessory (Diamond/ZnSe). The spectra were obtained in the wavenumber range of 4000–550 cm^–1^, with a resolution of 4 cm^–1^, by accumulating 16 scans.^24^ The FTIR- ATR vibrational bands were identified based on the literature.^25^

### Differential Scanning Calorimetry (DSC)

The thermal characteristics of the samples (melting, crystallization, oxidation, and decomposition temperatures) were measured on a 204 F1 Phoenix DSC (Netzsch, Germany). The samples were weighed (4 mg) into a pierced-lid aluminum pan and analyzed under an N_2_ flow of 40 mL/min, as already described by Teixeira et al.^24^ First, the samples were heated from 20 to 130 °C to eliminate the sample’s thermal history.^26^ Then, a cooling step to −10 °C and a second heating step, from −10 to 500 °C, were performed at a constant rate of 10 °C/min. Only the transitions observed during the cooling and the second heating cycles were considered.

### Antioxidant Assays

#### DPPH Assay

For the 2,2′-diphenyl-1-picrylhydrazyl radical (DPPH^•^) microplate method, the free radical scavenging activity was determined spectrophotometrically and performed in a Greiner Bio-One transparent 96-well microplate (North Carolina). The method was assayed as described by Bobo-García et al.^27^ with some modifications described by Teixeira et al.^24^ Briefly, samples were prepared in different concentrations, namely, from 7.0 to 0.20 mg/mL in methanol. Absorbance variation at 515 nm after the addition of samples or standard was measured in a Synergy H1TM microplate reader (BioTek Instruments, Inc.). The inhibition capacity is expressed as IC50 values. IC50 (mg/mL) was calculated by eq 1(28)
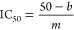
1The experiment was carried out in triplicate.

#### ABTS Assay

The 2,2-azino-bis-(3-ethylbenzothiazoline-6-sulfonate) (ABTS) assay was performed in a Greiner Bio-One transparent 96-well microplate (North Carolina), based on the inhibition by antioxidants of the absorbance of the radical cation ABTS^•+^ at 734 nm.^29^ The samples used in this method were the same prepared for the DPPH^•^ assay, and the analysis conditions were performed as Benteldjoune et al.,^30^ with some modifications described by Teixeira et al.^24^ Trolox equivalent antioxidant capacity (TEAC) was calculated using eq 2
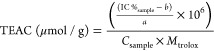
2The experiment was carried out in triplicate.

#### ORAC Assay

To perform the oxygen radical absorbance capacity (ORAC) microplate method, samples were prepared from a stock solution (2.5 mg/mL) with several subsequent sequential dilutions from 2.00 to 0.06 mg/mL in phosphate-buffered saline (PBS; 75 mM, pH 7.4). The stock solution was submitted to ultrasound (10 min) to improve the dissolution and then filtered using Macherey–Nagel 0.45 μm pore size Chromafil PET filters (Düren, Germany). The procedure was assayed as described by Dávalos et al.^31^ with some modifications evidenced by Teixeira et al.^24^ The fluorescence values were recorded every minute over the incubation period at 458 and 520 nm using a Synergy H1TM microplate reader (BioTek Instruments, Inc.), and the experiment was carried out in triplicate.

The antioxidant capacity was expressed as ORAC value in μmoles of Trolox equivalents (TE) per gram of the sample, and eq 3 was used,^32^ where the ORAC value (μmoles TE/L) corresponds to the *x* value obtained from Trolox linear regression (*y* = *mx* + *b*) by replacing *y* from AUC sample values. DF: dilution factor. L solvent/g sample: volume of the prepared mother solution/mass of the sample mother solution.
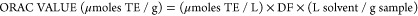
3

#### Total Phenolic Content Assay

The determination of the total phenolic content assay was performed as described by Papotti et al.,^33^ a modified Folin–Ciocalteu’s (FCr) method.^34^ Samples were prepared in ethanol at a stock concentration of 20 mg/mL and then diluted and mixed (50 μL) with 2.5 mL of diluted FCr 1:10 (v/v) and 2 mL of a hot Na_2_CO_3_ saturated solution. After incubation for 5 min at room temperature in the dark, the absorbance was determined at 760 nm in a UV-1900 UV–vis spectrophotometer (Shimadzu, Quioto, Japan). The total phenolic content was expressed as mg GAE/g of the sample (GAE: gallic acid equivalent) by comparison with a calibration curve with gallic acid standards from 25 to 800 μg/mL.

#### Cell Culture and Cytotoxicity against HaCaT

The human keratinocyte cell line HaCaT (CLS—Cell Line Services—300493) was kept in culture in Dulbecco’s modified Eagle medium (Thermo Fischer, Waltham, Massachusetts, EUA) supplemented with 10% fetal bovine serum (FBS) (Thermo Fischer, Waltham, Massachusetts, EUA) and 1% penicillin–streptomycin antibiotic (Thermo Fischer, Waltham, Massachusetts, EUA) at 37 °C, with 5% CO_2_ in a humidified atmosphere. The cytotoxicity of lipidic extracts on human immortal keratinocytes (HaCaT) was evaluated using a PrestoBlue (Thermo Fischer, Waltham, Massachusetts, EUA) assay according to the manufacturer’s instructions. Cells were seeded at 1 × 10^4^ cells/well in 96-well plates and exposed to lipidic extracts at different concentrations (2.50–0.16 mg/mL) diluted in Dulbecco’s modified Eagle medium (Thermo Fischer, Waltham, Massachusetts, EUA) for 24 h, in quadruplicates. Wells with media supplemented with lipidic extracts (without cells) were used to subtract a possible influence of the samples in the PrestoBlue fluorescence signal. Cells treated with 10% DMSO [Molecular Biology Grade, Merck (Darmstadt, Germany)] were used as a negative control. Afterward, the PrestoBlue reagent was added to the media and incubated for 2 h. The fluorescence signal was read in a Synergy H1 microplate reader (BioTek, Instruments, Inc.). Results are expressed in the percentage of metabolic inhibition in comparison to the control (cells without treatment). At least two independent experiments were performed.

#### Statistics

Results are reported as mean values ± standard deviation (SD). Data were first analyzed for normality distribution (i.e., Shapiro–Wilk). Levene’s test was applied to verify the homogeneity of the variances. Afterward, a one-way analysis of variance (ANOVA) test was applied with the Tukey post hoc test to determine differences within groups. For a two-group comparison, a Student’s t-test was assayed. The level of significance was set in general at 0.05. Analyses were performed with the aid of IBM SPSS Statistics software (28.0 version, Chicago).

For principal component analysis (PCA) and Heatmaps, the web-based tool suite Metaboanalyst (v5.0) was used (https://www.metaboanalyst.ca/).

## Results and Discussion

### Extract Yield and Monosaccharide Composition

Extraction parameters can greatly influence lipid yield. Accordingly, the results presented in this section were intended to establish the most suitable experimental conditions to achieve an optimal response between extract yield, purity (e.g., lower sugar content), and selective lipid composition.

**Figure 2:**
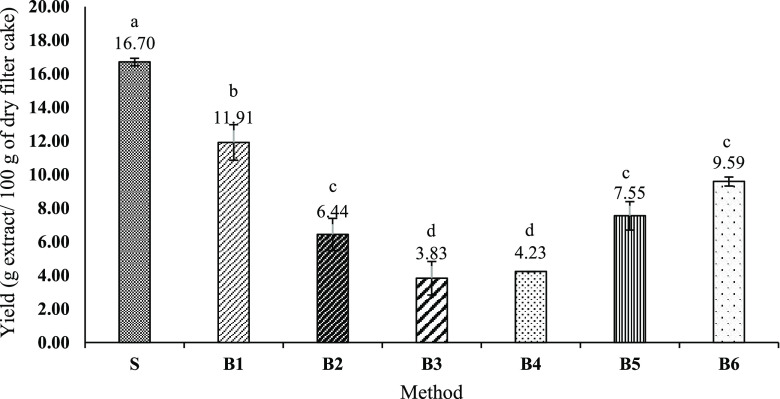
Extraction yields (g extract/100 g of dry filter cake) for the different tested methods. S: Soxtec; B: Boiling. Different superscript letters indicate statistically significant differences (*p* < 0.05).

The obtained results regarding isolation yields after using different extraction conditions on filter cake samples are shown in Figure 2. A yield value of 16.70 ± 0.22 g extract/100 g of dry filter cake was observed when assaying the S method (extraction performed using Soxtec). Moreover, the performance of this procedure was significantly higher than the other tested methods ranging from 11.91 ± 1.06 g extract/100 g (using B1) to 3.83 ± 1.00 g extract/100 g while assaying B3.

These differences in the isolation yield highlight the importance of both solvent proportion and temperature. Thus, while method S was carried out at 130 °C with a sample-to-solvent ratio of 1:20, procedures B1 and B2 were performed at 85 °C with ratios of 1:20 (B1) and 1:10 (B2). These latter results suggest that given the same temperature (85 °C for B1 and B2), the decrease of ethanol in the extraction mix harms the extract yield. Thus, for the same sample-to-solvent ratio, higher temperatures will increase extraction efficiency (i.e., S method). However, in order to understand what differences are associated with variations in yield, it is essential to study the composition of all extracts.

Since filter cake is a clarification byproduct of sugarcane juice, some monosaccharides may be retained and eventually recovered by the assayed processes of this work. Thus, further tests with mixtures of ethanol/water as well as aqueous washing of filter cake prior to the extraction attempted to increase the purity of the lipid extracts by decreasing sugar content.

**Figure 3:**
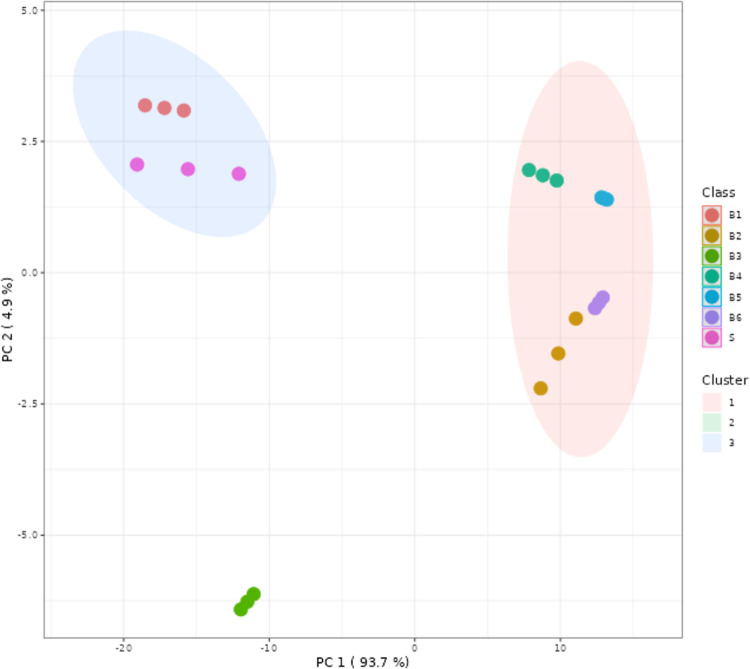
Principal component analysis (PCA) of monosaccharides quantification (by K-means clustering).

Saccharides such as rhamnose, arabinose, xylose, mannose, glucose, and galactose have already been identified in the water-soluble fraction of fruit waxes.^35^Table 2 presents the results concerning monosaccharides analyses (total, galactose, glucose, mannose, and arabinose) of the different samples. For all extracts, glucose was the main sugar detected. Accordingly, samples showing the highest total sugar contents were S (212.59 ± 19.67 mg/g), B1 (222.58 ± 7.48 mg/g), and B3 (195.77 ± 1.98 mg/g), which also presented the highest glucose concentrations. Figure 3 represents the principal component analysis (PCA) of monosaccharide quantification for all obtained extracts. Therefore, S and B1 extracts clustered together, which is indicative of the similarity in the monosaccharide profile of these samples.

**Table 2:** Monosaccharide Quantification (mg/g Extract) by GC-FID for Extracts Obtained by Different Extraction Methods^a^

extraction	total monosaccharides	galactose	glucose	mannose	arabinose
**S**	212.59^a^ ± 19.67	ND	203.15^a^ ± 26.23	9.45^c^ ± 1.58	ND
**B**_**1**_	222.58^a^ ± 7.48	ND	216.57^a^ ± 10.03	6.01^c^ ± 0.55	ND
**B**_**2**_	43.19^b^ ± 7.86	ND	30.85^b^ ± 6.79	12.34^b^ ± 2.87	ND
**B**_**3**_	195.77^a^ ± 1.98	9.27^a^ ± 0.43	156.19^a^ ± 3.69	24.21^a^ ± 0.28	6.11^a^ ± 0.18
**B**_**4**_	47.08^b^ ± 6.37	ND	47.08^b^ ± 6.37	ND	ND
**B**_**5**_	18.93^d^ ± 1.27	ND	18.93^b^ ± 1.27	ND	ND
**B**_**6**_	26.19^c^ ± 1.82	9.41^a^ ± 0.54	16.77^b^ ± 1.78	ND	ND

a Results expressed as mean ± SD (*n* = 3); ND, not detected. Different superscript letters in a row indicate statistically significant differences (*p* < 0.05).

Interestingly, method B3, which used an ethanol–water mixture (1:1) in a 1:20 sample/solvent ratio, was the only procedure able to recover galactose, glucose, mannose, and arabinose. This suggests that saccharides must be previously eliminated before proceeding with lipid isolation to increase the lipidic extract purity. Thus, to recover lipids from filter cake using ethanol, a previous extraction step was studied using water to eliminate monosaccharides.

The extraction with method B4, using a boiling prewash step with water before ethanol, led to isolation yields (i.e., 4.23 ± 0.01 g/100 g, Figure 2) similar to that of B3 (*p* ≥ 0.05). Also, the B4 method resulted in significantly lower total sugar content (47.08 ± 6.37 mg/g extract), Table 2, than S, B1, and B3. However, contents were close (*p* ≥ 0.05) to those of B2 (43.19 ± 7.86 mg total monosaccharides/g extract). These latter results may suggest that although ethanol can dissolve sugars, the amount of solvent used in the extraction is also a key factor in defining extract composition. The lowest total monosaccharide content was obtained when assaying methods B5 and B6 (such procedures are in the group of the ones using water prewash), and extraction yields were 7.55 ± 0.85 g extract/100 g and 9.59 ± 0.27 g extract/100 g, respectively (Figure 2). Moreover, the total sugar contents were 18.93 ± 1.27 mg/g extract and 26.19 ± 1.82 mg/g extract, respectively (Table 2).

In general, the S method extracts more sugars and leads to a more heterogeneous sample (i.e., it has no associated pretreatment and requires higher extraction temperatures), resulting in higher standard deviation in some of the assays analyzed for these extracts.

Thus, the reported data regarding extract yield and sugar composition for each wax highlights the impact of the assayed procedure. Hence, in order to select the most suitable isolation conditions, more information about the effect of each method on the lipid profile composition is crucial.

### Composition of Filter Cake Extracts by GC–MS

In the assayed chromatographic conditions, it was possible to identify different compounds such as fatty acids, fatty alcohols, phenolic acids, aldehydes, and phytosterols.

**Figure 4:**
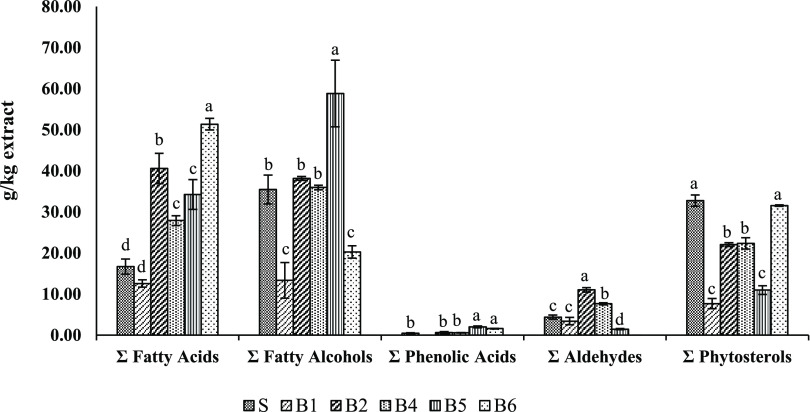
Main class composition (g/kg extract) of filter cake extracts obtained for the different extraction methods by GC–MS. S: Soxtec; B: Boiling. Different superscript letters indicate statistically significant differences (*p* < 0.05).

After evaluating the lipid composition of the extracts obtained with the different extraction methods by GC–MS (Table 3 and Figure 4), fatty acids, fatty alcohols, phenolic acids, aldehydes, and phytosterols were detected except for B3 extracts, where these compounds were not detected. In the B3 method, ethanol/water at a 1:1 ratio (v/v) was used as an extractant, and the obtained compositional results indicated that this process was not suitable for recovering lipophilic molecules. Thus, no fatty acids, fatty alcohols, phenolic acids, aldehydes, or phytosterols were detected by GC–MS.

**Table 3:** Composition (g Compound/kg Extract) of Filter Cake Extracts Obtained Using Different Extraction Methods by GC–MS^a^

compound ID	S	B_1_	B_2_	B_4_	B_5_	B_6_
coumaric acid	0.49^b^ ± 0.09	ND	0.62^b^ ± 0.27	0.60^b^ ± 0.05	2.04^a^ ± 0.24	1.60^a^ ± 0.08
**∑ phenolic acids**	**0.49**^**b**^ **± 0.09**	**ND**	**0.62**^**b**^ **± 0.27**	**0.60**^**b**^ **± 0.05**	**2.04**^**a**^ **± 0.24**	**1.60**^**a**^ **± 0.09**
palmitic acid	8.78^c^ ± 0.51	6.82^c^ ± 0.09	17.98^a^ ± 1.09	14.78^b^ ± 0.54	12.36^b^ ± 1.28	17.56^a^ ± 0.66
linoleic acid	3.08^c^ ± 0.61	1.88^c^ ± 0.44	10.96^a^ ± 0.82	4.69^c^ ± 0.18	7.55^b^ ± 0.85	13.87^a^ ± 0.26
oleic acid	3.07^c^ ± 0.42	2.44^c^ ± 0.04	7.76^a^ ± 0.60	4.86^b^ ± 0.30	5.31^b^ ± 0.54	9.18^a^ ± 0.02
stearic acid	0.82^c^ ± 0.01	0.94^c^ ± 0.01	1.84^a^ ± 0.28	1.57^b^ ± 0.10	1.49^b^ ± 0.13	2.12^a^ ± 0.12
octacosanoic acid	0.95^c^ ± 0.29	0.50^c^ ± 0.33	2.04^b^ ± 0.88	2.01^b^ ± 0.05	7.54^a^ ± 0.82	7.28^a^ ± 0.66
triacontanoic acid	ND	ND	ND	ND	ND	1.35^a^ ± 0.20
**∑ free fatty acids**	**16.70**^**d**^ **± 1.84**	**12.58**^**d**^ **± 0.91**	**40.58**^**b**^ **± 3.67**	**27.91**^**c**^ **± 1.17**	**34.25**^**c**^ **± 3.62**	**51.37**^**a**^ **± 1.40**
1-hexacosanol	2.40^b^ ± 0.26	0.95^c^ ± 0.23	3.12^b^ ± 0.01	3.68^a^ ± 0.13	4.32^a^ ± 0.37	1.34^c^ ± 0.16
1-octacosanol	27.76^b^ ± 2.42	10.35^c^ ± 2.94	28.96^b^ ± 0.41	27.23^b^ ± 0.30	39.27^a^ ± 5.49	15.68^c^ ± 1.10
1-triacontanol	3.78^b^ ± 0.46	1.41^c^ ± 0.72	4.24^b^ ± 0.02	3.60^b^ ± 0.11	8.91^a^ ± 0.97	2.27^b^ ± 0.17
1-dotriacontanol	1.51^b^ ± 0.38	0.63^b^ ± 0.44	1.84^b^ ± 0.04	1.45^b^ ± 0.02	6.33^a^ ± 1.30	0.99^b^ ± 0.05
**∑ fatty alcohols**	**35.45**^**b**^ **± 3.52**	**13.34**^**c**^ **± 4.33**	**38.16**^**b**^ **± 0.48**	**35.96**^**b**^ **± 0.56**	**58.83**^**a**^ **± 8.13**	**20.28**^**c**^ **± 1.48**
1-octacosanal	4.40^c^ ± 0.49	3.42^c^ ± 0.90	11.04^a^ ± 0.56	7.65^b^ ± 0.27	1.47^d^ ± 0.15	ND
**∑ aldehydes**	**4.40**^**c**^ **± 0.49**	**3.42**^**c**^ **± 0.90**	**11.04**^**a**^ **± 0.56**	**7.65**^**b**^ **± 0.27**	**1.47**^**d**^ **± 0.15**	**ND**
campesterol	5.95^a^ ± 0.40	1.31^c^ ± 0.12	3.82^b^ ± 0.07	3.98^b^ ± 0.27	3.83^b^ ± 0.45	6.26^a^ ± 0.06
stigmasterol	10.48^a^ ± 0.24	3.19^b^ ± 0.63	9.22^a^ ± 0.18	8.67^a^ ± 0.39	7.16^a^ ± 0.61	9.04^a^ ± 0.06
β-sitosterol	16.34^a^ ± 0.75	3.19^c^ ± 0.49	9.03^b^ ± 0.22	9.69^b^ ± 0.71	ND	16.26^a^ ± 0.18
**∑ phytosterols**	**32.77**^**a**^ **± 1.39**	**7.69**^**c**^ **± 1.24**	**22.07**^**b**^ **± 0.47**	**22.34**^**b**^ **± 1.37**	**10.99**^**c**^ **± 1.06**	**31.56**^**a**^ **± 0.18**

a Results expressed as mean ± SD (*n* = 3); ND, not detected. Different superscript letters in a row indicate statistically significant differences (*p* < 0.05).

Regarding the rest of the samples, as listed in Table 3, the B6 extract presented the highest concentration of total free fatty acids (FFA) (51.37 ± 1.40 g/kg). In general, palmitic acid was the main FFA present in all extracts (varying from 6.82 ± 0.09 g/kg in B1 to 17.98 ± 1.09 g/kg in B2).

According to the results, the lipid extracts contained interesting bioactive compounds previously described by other authors: coumaric acid (phenolic acid),^36,37^ 1-octacosanol (fatty alcohol),^11,38^ and β-sitosterol (phytosterols).^24,39^

It has been described that the phytochemistry of sugarcane wax includes phenolic acids that are considered antioxidant agents.^40^ In fact, coumaric acid was present in B5 extracts at 2.04 ± 0.24 g/kg and in B6 at 1.60 ± 0.08 g/kg.

Previous studies also reported that sitosterol is the main sterol in plants, but stigmasterol and campesterol also occur in nature.^41^ Furthermore, the S and B6 samples had the highest concentration of phytosterols, 32.77 ± 1.39 and 31.56 ± 0.18 g/kg, respectively.

The fatty alcohols that are present in sugarcane waxes are usually referred to as policosanols, octacosanol being one of the most characteristic alcohols.^42^ In the current study, 1-octacosanol was present in all extracts, varying from 10.35 ± 2.94 in B1 to 39.27 ± 5.49 g/kg in B5.

**Figure 5:**
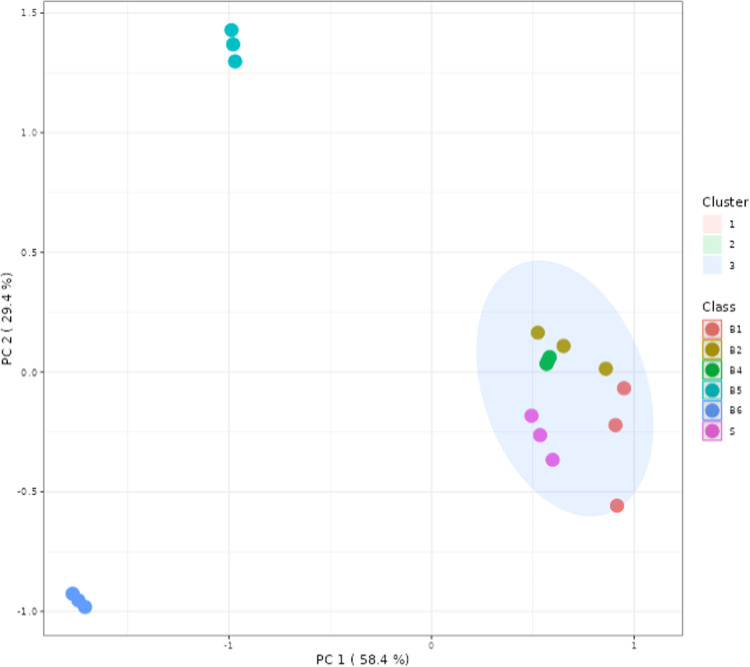
Principal component analysis (PCA) of GC–MS quantified compounds (by K-means clustering). Clusters: 1, 2, and 3. S: Soxtec; B: Boiling.

For the isolation of the abovementioned compounds (coumaric, phytosterols, and octacosanol) and to obtain higher extraction yield, the results suggest that the best boiling methods were the ones that included acidic water boiling followed by isolation with ethanol (B5 and B6 methods). Moreover, the procedure when the filter cake was dried prior to isolation (i.e., B5) showed the highest content of 1-octacosanol, whose amount varied in samples as follows: B5 ≥ B2 = B4 ≥ B6 = B1. In the case of coumaric acid, method B5 showed a trend toward higher levels than when assaying B6 (2.04 ± 0.24 vs 1.60 ± 0.08 g/kg, respectively), but these differences were not statistically significant (*p* ≥ 0.05). On the other hand, when the filter cake was not dried before and after boiling with water (i.e., B6), the concentration of phytosterols was higher than the other conditions. Interestingly, phytosterol contents in B5 extracts (10.99 ± 1.06 g/kg) were significantly lower than those in B6 (31.56 ± 0.18 g/kg). The only difference between these methods was that when using B5, the filter cake was dried prior to the boiling process, a higher time and energy-consuming method. Finally, principal component analysis (PCA) of the data obtained by GC–MS (Figure 5) revealed that B5 and B6 samples did not cluster with the other isolation methods, suggesting the compositional variation inherent to the acid boil pretreatment as well as the applied drying steps.

### Composition of Filter Cake Extracts by HPLC-ELSD

Samples were assessed by HPLC-ELSD, and results (Table 4) revealed the presence of some other lipid compounds, such as tocopherols, triglycerides, and glycolipids. As in the analyses performed by GC, samples obtained after extraction using the B3 method did not present these compounds.

**Table 4:** Analyses of the Contents of Tocopherol, Triglycerides, and Glycolipids by HPLC-ELSD (g/100 g Lipids) Obtained for the Different Extraction Methods^a^

compound ID	S	B_1_	B_2_	B_4_	B_5_	B_6_
**tocopherol**	ND	ND	ND	0.10^c^ ± 0.04	0.67^b^ ± 0.04	3.25^a^ ± 0.05
**triglycerides**	20.06^c^ ± 0.48	9.93^d^ ± 1.64	14.28^d^ ± 0.46	18.72^c^ ± 0.92	38.37^b^ ± 0.05	58.36^a^ ± 1.08
**glycolipids**	14.03^b^ ± 0.44	23.07^a^ ± 2.01	22.24^a^ ± 1.54	16.64^b^ ± 0.26	13.77^b^ ± 0.29	3.45^c^ ± 0.12

a Results expressed as mean ± SD (*n* = 3); ND, not detected. Different superscript letters in a row indicate statistically significant differences (*p* < 0.05).

The isolation conditions of the different methods greatly affected the composition of the extract. As shown in Table 4, methods B1 and B2 had the highest concentrations of glycolipids (*p* ≥ 0.05; 23.07 ± 2.01 g/100 lipids g for B1 extract, 22.24 ± 1.54 g/100 g lipids for B2 extract), while samples obtained using B6 showed the lowest levels (3.45 ± 0.12 g/100 g). On the other hand, for this latter extract, B6, levels of triglycerides were significantly higher than those found using other conditions and its variation was as follows: 58.36 ± 1.08 g/100 g lipids for B6 extract ≥ 38.37 ± 0.05 g/100 g lipids for B5 extract ≥20.06 ± 0.48 g/100 g lipids for S extract ≥ 18.72 ± 0.92 g/100 g lipids for B4 extract ≥14.28 ± 0.46 g/100 g lipids for B2 extract and ≥9.93 ± 1.64 g/100 g lipids for B1 extract.

As a result of the increasing commercial interest in health-promoting foods, tocopherol sources are widely studied.^43^ Aliphatic lipophilic compounds as tocopherols were previously quantified by del Río et al.^44^ in sugarcane straw and bagasse extracts, which reported 70 mg/kg of the dry straw sample. Indeed, tocopherol was identified by HPLC-ELSD mainly using the B6 extraction protocol (3.25 ± 0.05 g/100 g lipids).

**Figure 6:**
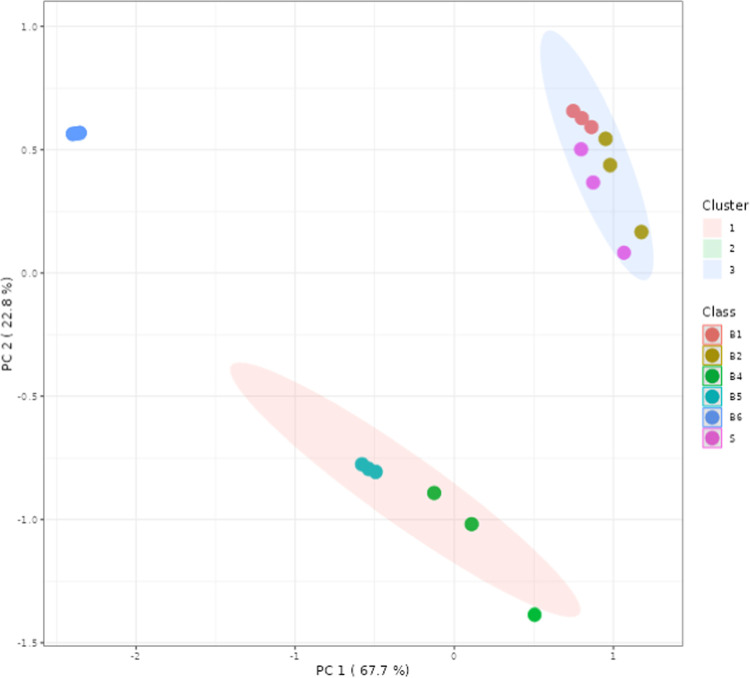
Principal component analysis (PCA) of HPLC-ELSD quantified compounds (by K-means clustering). Clusters: 1, 2, and 3. S: Soxtec; B: Boiling.

This analysis indicated that extracts obtained using the B6 method had a significantly lower concentration of glycolipids (3.45 ± 0.12 g/100 g lipids) and a higher concentration of triglycerides (58.36 ± 1.08 g/100 g lipids). This can be observed in Figure 6 by the PCA of HPLC-ELSD quantified compounds, where B6 does not cluster with the remaining extraction methods.

Overall, a distinct profile was observed by comparison of all extraction protocols. The used methodologies indicated that the B6 method was more efficient in extracting lipids such as fatty alcohols and phytosterols while maintaining lower total monosaccharide concentration. Hence, after analysis, the B6 method was found to be an efficient procedure for extracting sugarcane filter cake lipids, designated from this point as waxes. Method B6 was used to extract wax from different sugarcane crops, namely, Guariba (G) and Univalem (U), to explore the geographically related variation in the wax extract.

### Geographically Related Variation in Filter Cake Wax

Different batches of raw filter cake provided by Brazil (Guariba and Univalem) and collected in June 2020 were used to extract wax using the B6 method due to its effectiveness in extracting fatty alcohols and phytosterols while maintaining lower monosaccharide concentration and avoiding energy-consuming steps (i.e., sample drying). Extracts from Guariba and Univalem were labeled as B6_G and B6_U, respectively.

### Wax Yield and Monosaccharide Presence

By comparing B6_G and B6_U wax yields, it was possible to conclude that no significant differences (*p* ≥ 0.05) were detected between these two batches (9.97 ± 0.47 and 10.95 ± 1.36 g/100 g of dry filter cake, respectively). Table 5 contains the obtained results of monosaccharides quantification (mg/g extract) for these batches. Only galactose and glucose were identified, and the total concentration of monosaccharides was 31.73 ± 3.04 mg/g and 66.39 ± 3.98 mg/g for B6_U and B6_G, respectively. Results indicate that the lowest concentration in total monosaccharides was found for the Univalem batch, which presented a lower glucose concentration (15.66 ± 2.12 mg/g extract) than B6_G (46.04 ± 5.08 mg/g extract).

**Table 5:** Monosaccharide Quantification (mg/g Extract) of Different Filter Cake Wax Batches (B6_G and B6_U) Using the B6 Extraction Method^a^

batches	total monosaccharides	galactose	glucose
**B**_**6**_**_G**	66.39^a^ ± 3.98	20.35^a^ ± 0.49	46.04^a^ ± 5.08
**B**_**6**_**_U**	31.73^b^ ± 3.04	16.07^b^ ± 1.62	15.66^b^ ± 2.12

a Results expressed as mean ± SD (*n* = 3); ND, not detected. Different superscript letters in a column indicate statistically significant differences (*p* < 0.05).

### GC–MS Analysis

**Figure 7:**
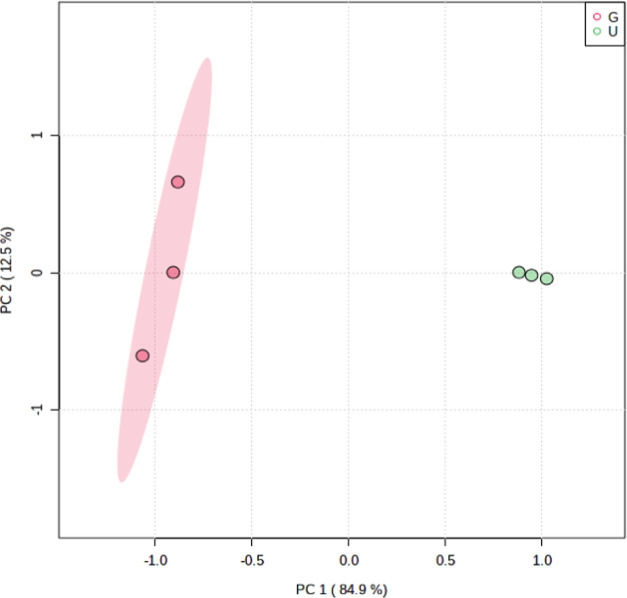
Principal component analysis (PCA) of GC–MS quantified compounds for the Guariba (G) and Univalem (U) batches (by K-means clustering).

Results concerning the analyses of B6_G and B6_U batches by GC–MS are presented in Table 6, and the principal component analysis (PCA) results are in Figure 7. Regarding the PCA, the two different batches are separated in PC1, which indicates their compositional variability. Although extracts presented some differences revealed by the GC–MS compositional profiling, free fatty acids, fatty alcohols, and phytosterols were the main compounds in both extracts (Table 6).

**Table 6:** Composition (g Compound/kg Extract) of Filter Cake Extracts Obtained Using Different Filter Cake Wax Batches (B6_G and B6_U) by the B6 Extraction Method by GC–MS^a^

compound ID	**B6_G**	**B6_U**
coumaric acid	1.88^a^ ± 0.26	0.61^b^ ± 0.04
**∑ phenolic acids**	1.88^a^ ± 0.26	0.61^b^ ± 0.04
palmitic acid	14.78^a^ ± 1.62	2.43^b^ ± 0.11
linoleic acid	16.99^b^ ± 1.85	21.98^a^ ± 0.34
oleic acid	9.27^b^ ± 1.01	14.43^a^ ± 0.08
stearic acid	0.79^b^ ± 0.14	2.04^a^ ± 0.17
octacosanoic acid	4.21^a^ ± 0.34	5.46^a^ ± 0.84
triacontanoic acid	0.68^b^ ± 0.15	1.29^a^ ± 0.32
**∑ free fatty acids**	46.72^a^ ± 3.09	47.62^a^ ± 1.17
1-hexacosanol	1.99^a^ ± 0.08	1.85^a^ ± 0.02
1-octacosanol	29.05^a^ ± 1.87	18.51^b^ ± 0.05
1-triacontanol	4.56^a^ ± 0.16	2.06^b^ ± 0.11
1-dotriacontanol	1.50^a^ ± 0.06	0.82^b^ ± 0.08
**∑ fatty alcohols**	37.09^a^ ± 1.85	23.23^b^ ± 0.11
1-octacosanal	0.90^b^ ± 0.03	2.86^a^ ± 0.51
**∑ aldehydes**	0.90^b^ ± 0.03	2.86^a^ ± 0.51
campesterol	5.76^a^ ± 0.01	5.99^a^ ± 0.01
stigmasterol	8.17^a^ ± 0.05	1.39^b^ ± 0.22
β-sitosterol	12.97^a^ ± 0.02	12.29^a^ ± 0.02
**∑ phytosterols**	26.90^a^ ± 0.07	19.66^b^ ± 0.19

a Results expressed as mean ± SD (*n* = 3); ND, not detected. Different superscript letters in a row indicate statistically significant differences (*p* < 0.05).

Total free fatty acids were quantified, and no significant differences were observed between batches (46.72 ± 3.09 vs 47.62 ± 1.17 g/kg extract, respectively, for B6_G and B6_U). However, significant differences in the concentration of linoleic acid in B6_G (16.99 ± 1.85 g/kg) and in B6_U (21.98 ± 0.34 g/kg) were found. The palmitic acid concentration was significantly lower in the B6_U batch (2.43 ± 0.11 g/kg) than that in B6_G (14.78 ± 1.62 g/kg; *p* < 0.05).

Fatty alcohols, mainly 1-octacosanol, were also identified in both batches. The B6_U batch presented lower (*p* < 0.05) concentration values of 1-octacosanol (18.51 ± 0.05 g/kg) than B6_G (29.05 ± 1.87 g/kg). The same behavior was found in total phytosterol content, which was higher (*p* < 0.05) for the B6_G (26.90 ± 0.07 g/kg) sample than B6_U (19.66 ± 0.19 g/kg). In both samples, β-sitosterol was the main phytosterol (12.97 ± 0.02 and 12.29 ± 0.02 g/kg for B6_G and B6_U, respectively), but campesterol (5.76 ± 0.01 and 5.99 ± 0.01 g/kg for B6_G and B6_U, respectively) and stigmasterol (8.17 ± 0.05 and 1.39 ± 0.22 g/kg for B6_G and B6_U, respectively) were also present in these waxes.

Coumaric acid concentration was higher (*p* < 0.05) in the B6_G batch, being almost 3-fold the concentration found in B6_U (1.88 ± 0.26 vs 0.61 ± 0.04 g/kg, respectively).

Octacosanol was the only aldehyde identified, and its content varied significantly among batches, as the concentration in the B6_U batch was 2.86 ± 0.51 g/kg and 0.90 ± 0.03 g/kg in B6_G.

The compositional differences observed for waxes resulting from geographically distinct crops can be supported by recent studies that indicated that leaf wax compounds as primary alcohols, alkanes, wax esters, aldehydes, and free fatty acids are independently regulated and contribute to phenotypic variation associated with epigenetic factors.^1,20^

### HPLC-ELSD Analysis

The results of the HPLC-ELSD analysis indicated that crops had significantly different tocopherol concentrations (*p* < 0.05; 2.27 ± 0.04 g/100 lipids for B6_G wax and 0.39 ± 0.05 g/100 g lipids for B6_U wax). The content of triglycerides was also higher for B6_G (32.27 ± 0.52 g/100 g lipids) than that for B6_U (24.27 ± 0.41 g/100 g lipids), and no major differences were found in the concentration of glycolipids for B6_G (11.68 ± 0.01 g/100 g lipids) when compared to B6_U (10.10 ± 0.05 g/100 g lipids).

### FTIR and DSC Analysis

Samples (B6_G and B6_U waxes) were analyzed on a PerkinElmer Paragon 1000 FTIR with the ATR accessory. The spectra were obtained in the wavenumber range of 4000–550 cm^–1^. The vibrational bands were identified based on the literature.^25^ The presence of the vibrational bands at 3363–3284 cm^–1^ (−OH stretching), 1712 cm^–1^ (−OH bending), 1168 cm^–1^ (−C–O asymmetric stretching), and 1050–1035 cm^–1^ (−C–O stretching), which are usually associated with alcoholic functional groups, are following the compositional GC–MS results that quantified fatty alcohols and phytosterols as two of the main families of compounds that constitute the analyzed waxes (B6_G and B6_U waxes).

Additionally, the vibrational bands at 2917 and 2849 cm^–1^ are related to the C–H stretching of −CH_2_ groups in aliphatic chains (asymmetric and symmetric, respectively); the vibrational bands at 1463 and 1377 cm^–1^ are associated with the C–H bending vibrations, respectively, of −CH_2_ and −CH_3_ groups, in aliphatic chains; and finally, the identification of the vibrational bands at 730 and 719 cm^–1^, both correlated to the rotational deformation of −CH_2_ groups in high aliphatic chains, indicates that the sample contains mostly aliphatic compounds of different functional groups, some of them with high aliphatic chains (≥C20). Moreover, these results were corroborated by the GC–MS analysis, which identified multiple aliphatic compounds within FFA, FOH, and aldehydes, namely, octacosanoic (C28) and triacontanoic (C30) acids, both with high aliphatic chains.

Filter cake waxes’ (B6_G and B6_U) thermal properties were also studied by differential scanning calorimetry (DSC) analysis (Table 7). Both waxes were solid at room temperature, and their melting and crystallization points were determined and revealed to be similar. The obtained crystallization temperature values were 54.3 and 58.5 °C for B6_G and B6_U, respectively. Concerning their melting points, the measured values were 65.4 °C and 67.8 °C for B6_G and B6_U, respectively. The enthalpy values associated with these transitions were similar enough to indicate that the amount of sample that melts and crystallizes is the same, suggesting that none of it degenerates during these transitions. Additionally, their high decomposition temperatures, 402.3 and 393.1 °C for B6_G and B6_U, respectively, associated with their high decomposition enthalpy values (258.2 J/g for B6_G and 384.7 J/g for B6_U) were a good indicator of their thermal stability.

**Table 7:** DSC Analysis of Different Filter Cake Wax Batches (B6_G and B6_U) Using the B6 Extraction Method

	temperature (°C) (|Δ*H*| (J/g))		
DSC	crystallization	melting	decomposition
**B**_**6**_**_G**	54.3 (57.7)	65.4 (63.0)	402.3 (258.2)
**B**_**6**_**_U**	58.5 (67.0)	67.8 (87.7)	393.1 (384.7)

### Antioxidant Activity and Biocompatibility on Keratinocytes

**Figure 8:**
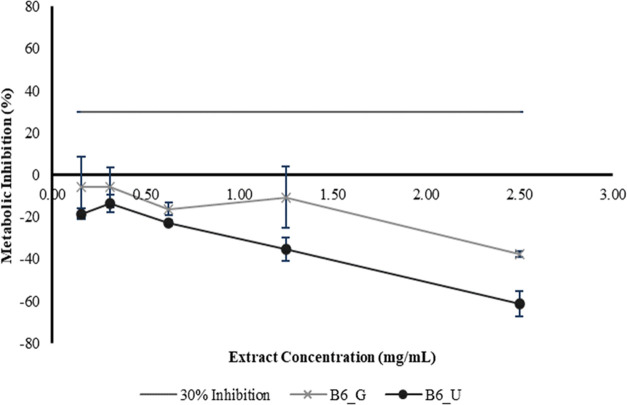
Cytotoxicity on the HaCaT cell line of different filter cake batches (B6_G and B6_U) using the B6 extraction method.

Studies regarding antioxidant properties (Table 8) and biocompatibility (Figure 8) on cultured human keratinocytes (HaCaT) were performed to verify if there were differences between Guariba (B6_G) and Univalem (B6_U) batches.

**Table 8:** Antioxidant (DPPH, ABTS, ORAC, and Total Phenolic Content) Analysis of Different Filter Cake Wax Batches (B6_G and B6_U) Using the B6 Extraction Method^a^

	DPPH	ABTS		ORAC	total phenolic content
antioxidant	IC_50_ (mg/mL)	IC_50_ (mg/mL)	TEAC (μmol/g)	(μmol TE/g)	(mg GAE/g)
**B**_**6**_**_G**	6.64^a^ ± 0.16	4.90^a^ ± 0.04	59.40^a^ ± 3.40	112.45^b^ ± 6.65	21.66^a^ ± 0.50
**B**_**6**_**_U**	6.07^a^ ± 0.01	4.40^a^ ± 0.01	56.10^a^ ± 2.70	230.36^a^ ± 16.95	23.05^a^ ± 1.22

a Results expressed as mean ± SD (*n* = 3); ND, not detected. Different superscript letters in a row for statistically significant differences (*p* < 0.05).

It is established that sugarcane rind phytometabolites that are responsible for its potential antioxidant activity can vary significantly among cultivated varieties. These compounds include anthocyanins, carotenoids, and terpenoids, which are positively correlated to sugarcane antioxidant capacity.^13,37^ After determining the optimal extraction process and quantification of lipophilic compounds, the antioxidant activity using DPPH, ABTS, and ORAC assays was evaluated for B6_G and B6_U waxes, and the results are shown in Table 8.

For the DPPH antioxidant assay, the IC_50_ values were 6.64 ± 0.16 and 6.07 ± 0.01 mg/mL for B6_G and B6_U, respectively (Table 8). The values obtained for the ABTS assay corroborate the previous results of DPPH scavenging activity, where no significant differences were observed between samples. However, the ORAC values indicated that the B6_U batch presented higher antioxidant activity (230.36 ± 16.95 vs 112.45 ± 6.65 μmol TE/g for B6_U and B6_G, respectively). Differences in the solubility of the obtained extracts for B6_U and B6_G batches were observed, the Univalem batch being more soluble in PBS, the solvent used in the ORAC method. This can be suggested as an explanation for the obtained differences in antioxidant activity for both samples when performing the ORAC assay. Nevertheless, no significant differences were found when performing the analysis on total phenolic content (21.66 ± 0.50 vs 23.05 ± 1.22 mg GAE/g for Guariba and Univalem, respectively).

The antioxidant activity of sugarcane samples has been proved elsewhere and is correlated to the richness of these samples in flavonoids, phytosterols, and fatty alcohols.^1,45−47^ Previous studies indicated that the TEAC values of sugarcane extracts obtained with 95% ethanol exhibited higher antioxidant activity (approx. 81.18 mg TE/g) than when using lower ethanol percentages.^45^ The antioxidant activity performed by DPPH (IC_50_ values) of sugarcane byproducts can range between 63 and 1000 μg/mL, which is relatively higher than the obtained values.^47^ Furthermore, the total phenolic content in sugarcane molasses extract was found to be 25.5 mg GAE/g elsewhere, which was similar to the obtained values in the current study.^48^

The cytotoxicity of the B6 resulting waxes was tested on the human immortal keratinocyte cell line (HaCaT) to evaluate its biocompatibility. Hence, in Figure 8, it is possible to observe that both samples (B6_U and B6_G) seem to be biocompatible on HaCaT at 2.5 mg/mL. The composition of these waxes, as well as their antioxidant and noncytotoxic outcomes, can be used in several cosmetic formulations as an alternative to the well-commercialized waxes.^49^

## Conclusions

Innovative applications for phytochemicals derived through environmentally friendly extraction processes require cutting-edge separation and identification methods based on multi-omics strategies.^6^ The determination of the lipophilic metabolite profile of sugarcane byproducts allows an extensive plant evaluation to predict its structural and metabolism-related functions.^6,8,46,50^

Filter cake samples were subjected to different ethanolic lipid extraction protocols and varying sample pretreatments including pH value and drying steps, temperature, and sample/solvent proportion. By avoiding sample drying before boiling with water and afterward ethanol (i.e., B6), the concentration of phytosterols was maximal. The obtained B6 extract yield was 9.59 ± 0.27 g/100 g of dry filter cake, and concentrations of fatty alcohols and phytosterols were 20.28 ± 1.48 and 31.56 ± 0.18 g/kg extract, respectively. Lower total monosaccharide concentration (26.19 ± 1.82 mg/g extract) was also the characteristic of this B6 resulting wax. This method was selected to extract wax from different sugarcane crops (Guariba and Univalem) and explore the geographically related variation in wax. Thus, the lowest obtained concentration in total monosaccharides was found for the Univalem crop. Fatty alcohols, mainly 1-octacosanol, were also identified in all batches, but the Univalem batch presented lower concentration values of 1-octacosanol than Guariba. In vitro free radical scavenging assays were applied to access the potential of sugarcane byproducts.^6,51^ The antioxidant values obtained for the ABTS assay corroborated the pattern observed in the DPPH assay, where no significant differences occurred. Hence, the ORAC values indicated that the Univalem batch presented higher antioxidant activity. Overall, the crop’s location influenced the wax composition, but the antioxidant activity was uncompromised, as well as its thermal properties. Hence, here, we demonstrate the suitability of wax production from industrial wastes using the B6 method, independent of *S. officinarum* L. cultivar location and without influencing the antioxidant performance. In fact, other studies related the richness of sugarcane in phenolic compounds (e.g., coumaric acid), as well as in phytosterols (e.g., β-sitosterol), to its antioxidant, antimicrobial, and anti-inflammatory properties.^10,52^ Consequently, this research work suggests that lipid extraction procedures do not require the usage of hazardous solvents, and a decrease of sample drying steps as well as a 90% ethanol recovery was achieved, allowing to bypass higher energy requirements and accomplishing better compositional performance.

## Abbreviations

FC: filter cake
TG: triglycerides
FFA: free fatty acids
FOH: fatty alcohols
DCM: dichloromethane
EtOH: ethanol
TEA: triethylamine
BSTFA: *N*,*O*-bis(trimethylsilyl)trifluoroacetamide with 1% trimethylchlorosilane
TMS: trimethylsilyl derivatives
HDL: high-density lipoprotein
LDL: low-density lipoprotein
FCr: Folin–Ciocalteu’s phenol reagent
ORAC: oxygen radical absorbance capacity
DPPH: 2,2′-diphenyl-1-picrylhydrazyl
ABTS: 2,2′-azino-bis(3-ethylbenzothiazoline-6-sulfonic acid)
PBS: phosphate-buffered saline
FBS: fetal bovine serum
DMSO: dimethyl sulfoxide
